# Peptide-Tetrapyrrole Supramolecular Self-Assemblies: State of the Art

**DOI:** 10.3390/molecules26030693

**Published:** 2021-01-28

**Authors:** Paolo Dognini, Christopher R. Coxon, Wendel A. Alves, Francesca Giuntini

**Affiliations:** 1 School of Pharmacy and Biomolecular Sciences, Byrom Street Campus, Liverpool John Moores University, Liverpool L3 3AF, UK; P.Dognini@ljmu.ac.uk; 2 Institute of Chemical Sciences, School of Engineering and Physical Sciences, Heriot-Watt University, Edinburgh AH14 4AS, UK; C.Coxon@hw.ac.uk; 3 Centro de Ciências Naturais e Humanas, Universidade Federal do ABC, Santo André, SP 09210-380, Brazil; wendel.alves@ufabc.edu.br

**Keywords:** peptide, tetrapyrrole, porphyrin, phthalocyanine, corrole, self-assembly, co-assembly, nanoarchitecture, supramolecular structure

## Abstract

The covalent and noncovalent association of self-assembling peptides and tetrapyrroles was explored as a way to generate systems that mimic Nature’s functional supramolecular structures. Different types of peptides spontaneously assemble with porphyrins, phthalocyanines, or corroles to give long-range ordered architectures, whose structure is determined by the features of both components. The regular morphology and ordered molecular arrangement of these systems enhance the photochemical properties of embedded chromophores, allowing applications as photo-catalysts, antennas for dye-sensitized solar cells, biosensors, and agents for light-triggered therapies. Chemical modifications of peptide and tetrapyrrole structures and control over the assembly process can steer the organization and influence the properties of the resulting system. Here we provide a review of the field, focusing on the assemblies obtained from different classes of self-assembling peptides with tetrapyrroles, their morphologies and their applications as innovative functional materials.

## 1. Introduction

Nature’s supramolecular machineries are a constant source of inspiration for the development of new smart materials, drugs and miniaturized tools for everyday life. Photosynthesis is perhaps the most striking example of a natural process carried out by assembled bio-macromolecules (i.e., photosystem 1 and 2 protein complexes) acting as dynamic templates for the nanoscale organization of tetrapyrrole chromophores (i.e., chlorophyll a and b) for light harvesting, electron transfer, and energy conversion [1,2]. Nature recurred repeatedly to the association of proteins with “the pigments of life” to achieve systems that can transport oxygen and catalyze reactions for the detoxification of oxygen radicals or the elimination of xenobiotics (e.g., heme in cytochrome, hemoglobin, and peroxidase) [3]. The quest to produce artificial analogues of such elegant and efficient machineries led to extraordinary advances in the understanding of supramolecular systems based on proteins and tetrapyrroles. Controlling the assembling process of proteins to achieve tetrapyrrole-containing complexes able to accomplish the desired functions is no trivial feat [4]: The pursuit to simplify the target systems inspired scientists to explore the potential of synthetic peptides as more easily accessible building blocks [5]. This opened the way to a new class of functional peptide-based materials in which the assembling behavior of particular peptides is exploited to achieve ordered distribution of natural and synthetic tetrapyrroles at nanoscale, to harness their photochemical and photophysical properties. In this review, we will describe these systems, highlighting, wherever possible, the driving force(s) that promote the assembly and the influence of each component in the process. The behavior and features of the self-assembled supramolecular architectures compared to the individual components or non-organized structures will also be presented.

### 1.1. Assembly of Peptides and Tetrapyrroles

Self-assembly occurs when single molecular species spontaneously establish specific and repeated interactions with each other, giving rise to an organized supramolecular structure [6]. This process is often initiated by conditions/stimuli that trigger the formation of noncovalent interactions between the molecules in the system (e.g., hydrogen bonding, electrostatic and/or hydrophobic interactions, π–π stacking, and van der Waals forces), resulting in a reversible long-lived mid- or long-range organization. The ordered nature of assemblies makes them different from aggregates, which are clusters of molecules or particles in which no long-range order can be identified. The term supramolecular assembly is commonly used to indicate larger structures, typically with rod-, sheet-like, or spherical shape, and dimensions ranging from nm to μm scale. The term co-assembly denotes a self-assembly process where two or more components form the organized structure.

Self-assembling peptides are a heterogeneous class of naturally occurring and synthetic peptides with a length ranging from 2 to 50 amino acid residues. The ability to form long-range ordered supramolecular architectures in given environment has made these species the focus of attention in various scientific fields [7,8]. Self-assembling dipeptides [9], peptide amphiphiles [10], bolaamphiphiles [11,12], as well as cyclic [13] and collagen-like peptides [14] form α-helix, β-sheet, and/or surfactant-like assemblies that further organize into nanoarchitectures with various morphologies. These species showed potential for applications as bioactive materials [15], biosensors [16], drug delivery systems [17], 3D scaffolds for cell culture [18], electronic devices [19], supports for tissue regeneration [20] and bio-printing [21] to name but a few. For reviews on self-assembling peptides, the reader is referred to Luo et al., Rad-Malekshahi et al., or Gerbelli et al. [22,23,24].

**Figure 1:**
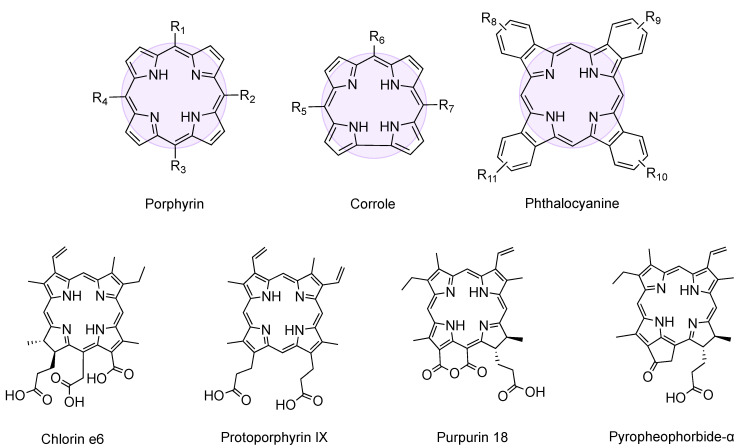
General structure of porphyrin, corrole, and phthalocyanine, whose tetrapyrrolic ring is highlighted. Molecular structure of chlorin e6, protoporphyrin IX, purpurin-18, and pyropheophorbide-α.

Tetrapyrroles are a family of cyclic aromatic species that includes, amongst others, porphyrins, phthalocyanines and corroles (Figure 1). These large aromatic macrocycles are characterized by high chemical stability and they form complexes with a variety of metal ions [25]. The strong absorption in the UV and visible wavelengths, small HOMO-LUMO gap and the efficient emission displayed by many members of this family are some of the photophysical features that make these species unique [26]. The high excited triplet state yield and the ability to transfer electrons to neighboring chemical species has afforded tetrapyrroles an advantage in applications as agents for antimicrobial or anticancer photodynamic therapy [27,28], optical sensors or probes [29], catalysts [30], single-molecule magnets [31], and organic semiconductors for solar cells technologies [32]. Tetrapyrroles display a strong aggregative behavior in solution [33,34,35]. The formation of J-aggregates (side-by-side) or H-aggregates (face-to-face) depends on the tetrapyrrole substitution and environmental conditions and results in a transition dipole moment that strongly influences the electronic properties and photo-response of the chromophore. In porphyrins, the aggregation into J-type aggregates results in red-shifts of the absorbance bands, whereas H-aggregates cause a blue-shift. Phthalocyanine and corroles also exhibit similar behaviors [36,37,38].

The potential of self-assembling peptides as biocompatible, biodegradable and synthetically accessible species to impart supramolecular order to tetrapyrrole arrays fuelled numerous studies in the last decade. The introduction of suitable functional groups on the tetrapyrrole ring, usually on the *meso* position in porphyrins and corroles and on the condensed benzene rings in phthalocyanines, allows the conjugation to self-assembling peptides, affording conjugates that adopt the desired ordered architectures in specific environments. Frequently, the peptide changes its assembling behavior as a consequence of its conjugation with the tetrapyrrole and the final supramolecular arrangement is determined by the assembling behavior of both the peptide and tetrapyrrole moieties. The co-assembly of non-covalently linked tetrapyrroles and peptides has also been the subject of numerous studies. In this case, the two components are separate and interact via noncovalent interactions: The peptide generally acts as a scaffold and most of the time no significant changes in its supramolecular morphology result from the presence of the tetrapyrrole. In these systems the peptide–porphyrin ratio can be altered, allowing control of the chromophore density and consequent modulation of the system’s photophysical and photochemical properties. Notably, most of the supramolecular assemblies reviewed hereafter have properties ascribable to the formation of tetrapyrrole J-aggregates within the architecture, while H-aggregation seems to be less common.

**Figure 2:**
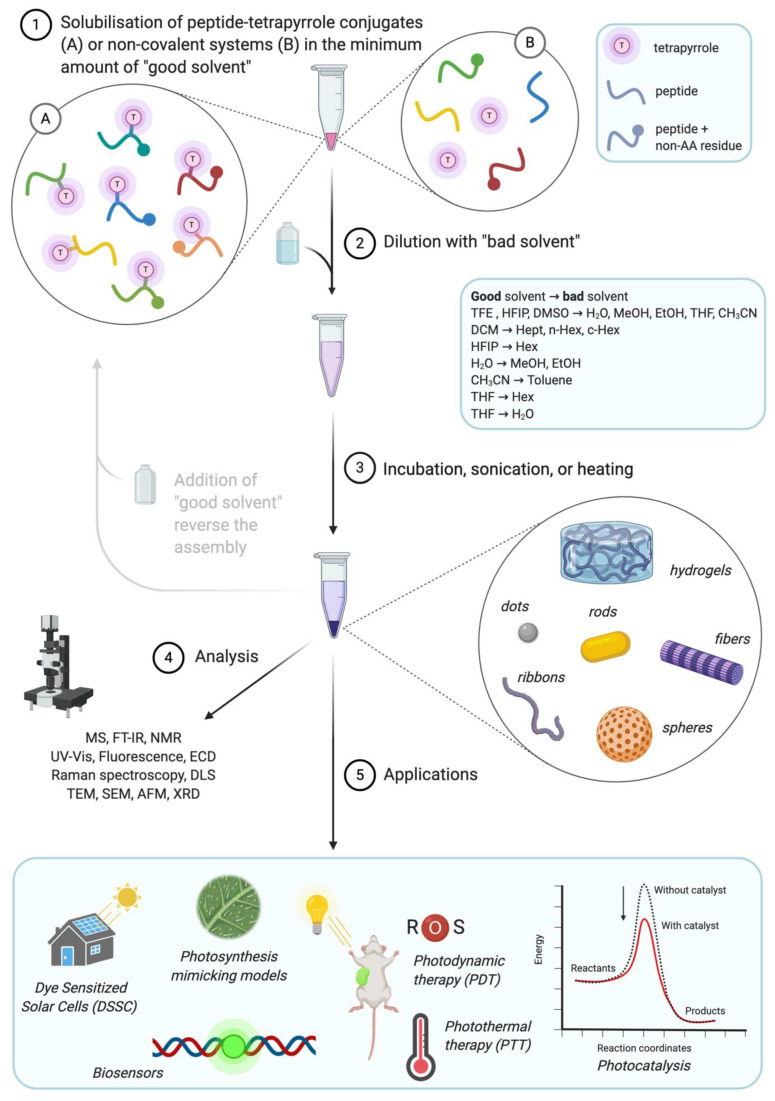
Overview of solvent switch preparation steps (1–3), usually employed analytical techniques (4) and reported applications (5) of peptide-tetrapyrrole supramolecular assemblies. Created with BioRender.com. TFE = trifluoroethanol; HFIP = hexafluoroisopropanol; DMSO = dimethyl sulfoxide; THF = tetrahydrofuran; DCM = dichloromethane; Hept = heptane; Hex = hexane; MS = Mass Spectrometry; FT-IR = Fourier-Transformed Infrared Spectroscopy; NMR = Nuclear Magnetic Resonance; ECD = Electronic Circular Dichroism; DLS = Dynamic Light Scattering; TEM = Transmission Electron Microscopy; SEM = Scanning Electron Microscopy; AFM = Atomic Force Microscopy; XRD = X-Ray Diffraction.

Figure 2 illustrates the general approach for the formation of peptide–tetrapyrrole supramolecular assemblies, as well as their analysis and applications. The most common procedure to induce self-assembly is based on the “good–bad solvent” or “solvent switch” method, although changes to pH and temperature are also used. In this work, we will review the recent advancement in the field of self-assembling systems made by tetrapyrroles covalently linked or associated to short self-assembling dipeptides (e.g., diphenylalanine), longer sequences, cyclic peptides, and peptide bearing moieties of other nature. Their molecular structures are listed in Table 1, Table 2, Table 3, Table 4 and Table 5 while Table S1 also summarizes morphologies and applications. Systems comprising tetrapyrroles and proteins, single amino acids or amino acid polymers (e.g., polylysine) [39,40,41,42,43,44] have not been considered.

## 2. Diphenylalanine

**Figure 3:**
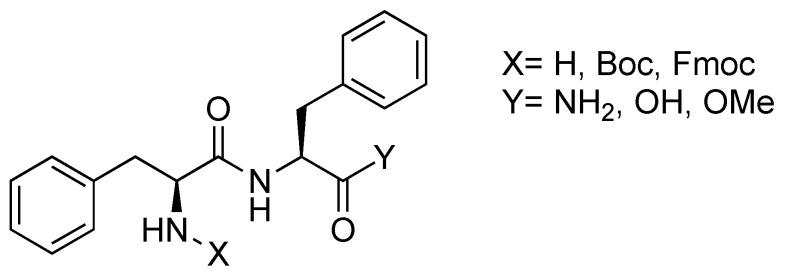
l,l-diphenylalanine structure.

The self-assembling behavior of diphenylalanine (Phe-Phe or FF, Figure 3) has been the subject of interest since it was identified as the shortest recognition motif for the aggregation of β-amyloid polypeptides [45,46,47,48].

Reches and Gazit reported that FF self-assembles in nanotubes by dilution of a concentrated solution of dipeptide in hexafluoroisopropanol with water [19]. FF self-assemblies are relatively straightforward to obtain and display exceptional stability [49,50,51]: The supramolecular structures they adopt have been widely studied and exploited as biocompatible materials for numerous applications. Self-assembling structures comprising tetrapyrroles and FF have been developed, including conjugates, complexes and noncovalent systems (Table 1).

**Table 1:** FF-tetrapyrrole conjugates, complexes and noncovalent assembling systems. Refer to Figure 1 and Table S1 for details on structure and further information. [X] = metal atoms coordinated by the tetrapyrrole ring; Boc = tert-butyloxycarbonyl; Fmoc = fluorenylmethoxycarbonyl; BODIPY = 4,4-difluoro-4-bora-3a,4a-diaza-s-indacene.

Entry	Peptide	Tetrapyrrole	Interaction (Linker/Bond)	Ref.
**1**	(**a**) FF (**b**) Boc-FF (**c**) Fmoc-FF	Porphyrin R_1_ = 4-aminophenyl R_2_, R_3_, R_4_ = phenyl	Covalent (amide)	[52,53]
**2**	FF-OMe	Porphyrin R_1_ = 4-aminophenyl R_2_, R_3_, R_4_ = phenyl	Covalent (triazine)	[54]
**3**	Boc-FF	Porphyrin R_1_ = 3,4-diaminophenyl R_2_, R_3_, R_4_ = phenyl	Covalent (amide)	[54]
**4**	Boc-FF	Porphyrin [SnOH_2_] R_1_, R_2_, R_3_, R_4_ = phenyl	Metal coordination	[54]
**5**	(**a**) Fmoc-FF (**b**) FF-OMe	Corrole R_5_, R_7_ = pentafluorophenyl R_6_ = 4-aminophenyl	Covalent (**a**) amide (**b**) triazine	[54]
**6**	Boc-FF	Porphyrin R_1_ = 4-carboxyphenyl R_2_, R_4_ = mesityl R_3_ = 4-aminophenyl	Covalent (amide)	[55]
**7**	(**a**) FF (**b**) Boc-FF (**c**) Fmoc-FF	Porphyrin R_1_, R_2_, R_3_, R_4_ = 4-aminophenyl	Covalent (amide)	[56]
**8**	FF-BODIPY	Porphyrin R_1_ = 4-carboxyphenyl R_2_, R_3_, R_4_ = phenyl	Covalent (amide)	[56]
**9**	FF	Porphyrin R_1_ = 4-carboxyphenyl R_2_, R_3_, R_4_ = phenyl	Covalent (amide)	[57]
**10**	FF	Porphyrin R_1_ = 4-aminophenyl R_2_, R_3_, R_4_ = phenyl	Covalent (triazine)	[58]
**11**	FF	Porphyrin R_1_ = 4-aminophenyl R_2_, R_3_, R_4_ = phenyl	Covalent (glutaric acid)	[59]
**12**	FF	Phthalocyanine [Zn] R_8_, R_9_, R_10_ = H, R_11_ = OH	Covalent (butanoic acid)	[60,61]
**13**	FF-NH_2_·HCl	Chlorin e6	Noncovalent	[62]
**14**	FF	Porphyrin R_1_, R_2_, R_3_, R_4_ = hydroxyphenyl	Noncovalent	[63]
**15**	Fmoc-FF	Porphyrin [Zn]; [Sn] R_1_, R_2_, R_3_, R_4_ = 4-pyridyl	Noncovalent	[64]
**16**	Fmoc-FF	Porphyrin (**a**) R_1_, R_2_, R_3_, R_4_ = 4-carboxyphenyl (**b**) R_1_, R_2_, R_3_, R_4_ = 4-aminophenyl	Noncovalent	[65]
**17**	FF	Porphyrin R_1_, R_2_, R_3_, R_4_ = 4-sulfonatophenyl	Noncovalent	[66]
**18**	D-F-D-F-NH_2_	Porphyrin [Sn(OH)_2_] R_1_, R_2_, R_3_, R_4_ = 4-tolyl	Noncovalent	[67]
**19**	FF	Phthalocyanine [Zn] (**a**) R_8_, R_9_, R_10_, R_11_ = solketal (**b**) R_8_, R_9_, R_10_, R_11_ = glyceryl (**c**) R_8_, R_9_, R_10_ = solketal; R_11_= methyl (**d**) R_8_, R_9_, R_10_ = glyceryl; R_11_= methyl	Noncovalent	[68]

Charalambidis et al. obtained self-assembled nanospheres by diluting hexafluoroisopropanol solutions of conjugates **1a**–**c** with a polar solvent in a 2:8 ratio [52]. These nanostructures have solvent dependent sizes, regular shapes, and porous surfaces. Interestingly, the formation of spherical structures is reversible and was not observed when FF or porphyrin monomers were used, indicating that the assembling behavior is ascribable to the specific structure of the porphyrin-peptide conjugate. Karikis et al. studied the self-assembly of conjugates **2**, **3**, **4**, and **5a**,**b** to gain further insight on the photophysics of porphyrinoids aggregates [54]. The authors observed that corrole-FF conjugates **5a**,**b** yield nanospheres similar to those obtained with porphyrin-containing analogues and that corrole conjugates bearing only C or G do not self-assemble under the same conditions. These behaviors suggest a stronger influence of the peptide on the assembling process, considering the lower symmetry and stronger acidity and basicity of corroles compared to porphyrins. The photosensitizing ability of the porphyrin is maintained in the assemblies and their oxidation potential is suitable for electron transfer to the conduction band of the semiconductor (TiO_2_) [52]. Supramolecular self-assembled antennas of the Zn(II) complex of conjugate **1b** were formed on the surface of TiO_2_ electrodes to obtain a solid-state dye sensitized solar cell (DSSC) [55]. The assembly is triggered by conjugate **6**, anchored on the TiO_2_ surface through a carboxylate. Compared to the single component solid or liquid DSSC, the association between the two dipeptide–porphyrin conjugates significantly improves the photovoltaic performance of the device. The authors exploited the solvent-tunable morphology of these structures to develop an artificial antenna system that switches from an active to inactive state, depending on the solvent polarity [53]. In 1:1 dichloromethane/heptane solution, conjugate **1c** assembled into nanospheres that displayed an intense fluorescence and can potentially transfer energy to an energy trap. The system switched off in a less polar solvent (dichloromethane/heptane ratios of 3:7 and 2:8), where microfibrils and platelet tangles with strong excitonic couplets and completely quenched fluorescence are formed. Most recently, Nikoloudakis et al. conjugated four FF units to a porphyrin (conjugates **7a**–**c**) and synthesized conjugate **8**, which contains BODIPY as an additional chromophore [56]. Tetrasubstituted porphyrins self-assembled to give different morphologies (nanospheres, plaques, and microfibrils) depending on the protecting group on the peptide and solvent system. The same variables influenced the formation of spherical architectures or amorphous systems from conjugate **8**.

Conjugate **9** self-assembles into nanofibers, which then aggregate to form multi-porous and water-stable microspheres in hexafluoroisopropanol/toluene [57]. The broad-spectrum absorption and enhanced photoelectron transfer of the assembled system encouraged Tao et al. to explore it as a sunlight-sensitive antenna for artificial photosynthesis and as a component of Pt nanoparticles-based photocatalytic systems for NADH regeneration.

The morphology of the assemblies reported by Li et al. varies with conjugate **10** concentration: Clusters of nanosheets, “pumpkin-like” nanoparticles and nanospheres were observed after the addition of an increasingly concentrated hexafluoroisopropanol solution in *n*-hexane [58]. The authors confirmed that porphyrin J-aggregates can delocalize excited states within a self-assembling supramolecular architecture and promote electron transfer. Additionally, they specifically demonstrated the crucial role of the dipeptide for the self-assembling process, as no aggregates were observed in porphyrin-only samples.

Conjugate **11** self-assembles in dimethyl sulfoxide/water to afford stable nanodots that were studied by Zou et al. as photothermal therapy (PTT) and photoacoustic imaging (PAI) agents [59]. The formation of porphyrin J-aggregates in the dot core completely quenches fluorescence emission and ROS production, leading to a high light-to-heat conversion efficiency. The nanodots (20 to 100 nm in size) accumulate in tumor tissue and cause its photothermal ablation in vitro and in vivo following irradiation, while having negligible toxicity in the dark.

Zhao et al. achieved kinetic control of the self-assembling process of conjugate **12** to form thermodynamically stable nanofibrils instead of nanospheres by altering the dimethyl sulfoxide/water ratio (1:80 to 1:5) and increasing the assembly time [60]. The low optical activity of nanospheres and the intense nanofibrils circular dichroism (CD) pattern characteristic of a right-handed helical structure highlight the profound difference in supramolecular order between the two species. Li et al. studied conjugate **12** nanospheres as spatiotemporally coupled phototherapy agents [61]: Their peculiarity is the ability to switch, after interaction with the cell membrane, from an assembled structure applicable in PTT and PAI to peptide-phthalocyanine monomers with photodynamic therapy (PDT) and fluorescence imaging (FI) properties. The combination of these light-induced effects makes this system effective against tumor growth in vitro and in vivo.

Liu et al. modulated the co-assembly of chlorin e6 and FF (**13**) to obtain innovative nanoparticles with controllable size and chlorin payload for applications in anticancer PDT [62]. In these systems, the tetrapyrrole is both a drug and a component of the carrier, whose disassembly is triggered by pH changes, surfactant and enzymes. This happens when the nanoparticles are internalized by endocytosis, resulting in the release of chlorin. The assemblies exhibited enhanced cellular uptake and photo-cytotoxicity compared to stand-alone chlorin e6, efficient photodynamic activity in vitro and no dark toxicity.

Kim et al. reported the co-assembly of FF and tetrakis(4-hydroxyphenyl)porphyrin (**14**) [63]. Dilution of a hexafluoroisopropanol solution of the two species with phosphate buffer led to the formation of porphyrin J-aggregates on the surface of FF nanotubes and conferred light harvesting properties to the system. The deposition of Pt nanoparticles on the nanotubes increased the efficiency of the photoinduced electron separation and transfer, affording efficient biomimetic antenna system for visible light-driven NADH regeneration and redox enzymatic synthesis of l-glutamate. Kim et al. also achieved photocatalyzed water oxidation with a colloidal metal-oxide obtained from the co-assembly of **15 [64]**. In this case, the Fmoc-protected dipeptide gives a self-standing hydrogel composed of β-sheet nanofibers; irradiation with visible light triggered electron transfer between porphyrin J-aggregates which, in combination with IrO_2_, led to oxygen evolution from water. A dimethyl sulfoxide/water microfluidic-based method has also been employed by Li et al. to co-assemble systems **16a**,**b** into a hydrogel [65].

When FF and tetrakis(4-sulfonatophenyl)porphyrin (**17**) co-assemble in an acidic aqueous environment into an interconnected and self-supporting network of dipeptide and porphyrin nanorods (in a 2.3:1 molar ratio), this forms porous, multi-chambered and water-filled microspheres [66]. Fe(III) coordination impairs microsphere assembly as it precludes the initial formation of anionic porphyrin J-aggregates surrounded by cationic FF stacking. This colloidal supramolecular structure captures light when irradiated and is stable to photodegradation. The association of positively charged inorganic and organic species to the negatively charged porphyrins inside the microspheres encourages its applications as catalyst for photochemical transformations (e.g., redox reactions).

The co-assembly of the d-enantiomer of FF with a tin porphyrin (**18**) was achieved with a solvothermal method by Parayil et al. [67] and peptide nanoribbons with lipophilic metalloporphyrins dispersed into the cavities of their porous surface were obtained. The emission spectra of these highly fluorescent and thermally stable structures are specifically affected by DNA binding, making them potential biocompatible DNA sensors. 

Souza et al. reported photosensitizer carriers obtained from the co-assembly of **19a**–**d** in hexafluoroisopropanol/methanol/water [68]. Long, square, and sharp-edged anisotropic structures with hexagonal symmetry present interstices that host Zn(II) phthalocyanine and variable surface roughness (terraces, granules, striations, and grooves) that depends on the tetrapyrrole unit substitution. Compared to phthalocyanine species alone, these peculiar assemblies have high stability, low toxicity, strong fluorescence, and singlet oxygen production, enhanced accumulation in MCF-7 cells and can thereby promote more efficiently necrotic cell death upon irradiation. 

The presence of the FF is paramount for the self-assembly of the systems reviewed in this section. The ability of the peptide to establish hydrogen bonds and stacking interactions provides the driving forces for the FF self-assembly but the synergic influence of large π-conjugated tetrapyrroles can promote the formation of the assembly and affect its shape, leading to different morphologies and spatial arrangements. The works published show that the self-assembly of these systems tolerates considerable variations of the tetrapyrrole structure and is not affected by the conjugation of the dipeptide via N- or C-terminus. The influence of the protecting groups will be discussed in detail in Section 5.2.

## 3. Other Dipeptides

**Figure 4:**
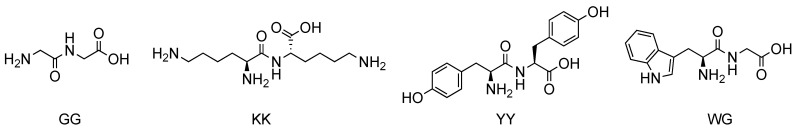
Chemical structures of GG, KK, and YY dipeptides.

FF is perhaps the best-characterized self-assembling peptide, yet other dipeptides such GG, KK, YY, and WG (Figure 4) are capable of spontaneously generate supramolecular structures in the presence of porphyrins (Table 2).

**Table 2:** Dipeptide-tetrapyrrole conjugates and noncovalent assembling systems. Refer to Figure 1 and Table S1 for details on structure and further information.

Entry	Peptide	Tetrapyrrole	Interaction (Linker/Bond)	Ref.
**20**	GG	Porphyrin R_1_ = 4-carboxyphenyl R_2_, R_3_, R_4_ = 3-methoxyphenyl	Covalent (amide)	[69]
**21**	KK	Porphyrin R_1_, R_2_, R_3_, R_4_ = 4-sulfonatophenyl	Noncovalent	[70,71]
**22**	YY	PorphyrinR_1_ = 1,3-bis(octyloxy)phenyl R_3_ = 4-(bromomethyl)phenyl R_2_, R_4_ = H	Covalent (ether)	[72]
**23**	WG	Porphyrin R_1_, R_3_ = 4-carboxyphenyl R_2_, R_4_ = phenyl	Covalent (amide)	[73]

Teixeira et al. showed that conjugate **20** organizes from nonspecific H- and J-aggregates into larger ordered supramolecular structures upon the addition of small volumes of ethanol to aqueous solutions [69]. The system rearranges to form completely dispersed supramolecular spheres and rods, depending on the monomer concentration and the percentage of ethanol added.

The co-assembly of positively charged dipeptide KK with a negatively charged porphyrin (**21**) in acidic aqueous solution afforded nanorod-shaped J-aggregates that aligned to form long fiber bundles, ordered at the microscale level [70,71]. This organization confers stability to the system, as well as compatibility with different ions. Other than showing the evolution of the assembly over time, CD of fiber bundles detected a much more intense Cotton effect than in J-aggregates alone, indicating that KK induces an amplification of the chiral supramolecular arrangement of the porphyrins. The light-induced oxidation of iodide to triiodide and the photocatalytic synthesis of Pt nanowires and nanospheres are a demonstration of the improved photoelectronic properties. Light harvesting, charge separation and hydrogen evolution can also be achieved after self-mineralization of the fibers with TiO_2_/Pt, in a system that mimics the architectures and functionalities of chlorophylls in green sulfur bacteria.

Kim et al. synthesized a conjugate comprising two hydrophobic porphyrins linked by the dipeptide YY, which was subsequently cyclized into the corresponding diketopiperazine derivative (conjugate **22**) [72]. The appearance of a strong and broad CD band around 375 nm highlights H-aggregates formation and concentration-dependent self-assembly of this specie in toluene. The resulting micrometric fibers or toroidal architectures display potential light harvesting properties.

Sun et al. presented a pH-sensitive nanomaterial for PDT self-assembled from a porphyrin bearing two dipeptides (**23**) [73]. The distinctiveness of this supramolecular architecture is the ability to re-assemble from spheres to fibers in the acidic environment typical of tumor tissues. The fibrillar structure enhances ROS production and long-term retention that, combined with selective accumulation at the tumor site, results in notable in vitro phototoxicity against cancer cells and in vivo anti-tumor activity without off-target side effects.

Given the lack of intrinsic self-assembling behavior of these dipeptides, the role that the tetrapyrrole can play in enabling supramolecular organization stands out. The presence of the dipeptide, however, imparts long-range order and chirality to the assembly that would not be achievable through the porphyrin-driven aggregation.

## 4. Medium and Long Peptides

Increasing the length of the peptide makes its synthesis more challenging and the peptide–porphyrin system more complex. However, conjugates and noncovalent systems containing longer peptide sequences were successfully obtained, allowing fine-tuning of the behavior of the resulting supramolecular structure through variation in the structure and tetrapyrrole conjugation strategies. The self-assembling peptide sequences of more than two amino acids combined with tetrapyrroles are summarized in Table 3.

**Table 3:** Medium/long peptide-tetrapyrrole conjugates, complexes and noncovalent assembling systems. Refer to Figure 1 and Table S1 for details on structure and further information. [X] = metal atoms coordinated by the tetrapyrrole ring; 4-Pal = 4-pyridylalanine.

Entry	Peptide	Tetrapyrrole	Interaction (linker/bond)	Ref.
**24**	Ac-CKVKV-NH_2_	Porphyrin (**a**) R_1_ = 4-bromoacetamidophenyl (**b**) R_1_ = 2-bromoacetamidophenyl R_2_, R_3_, R_4_ = 4-tolyl	Covalent (thioether)	[74,75]
**25**	Ac-CKVSVKV-NH_2_	Porphyrin R_1_ = 4-bromoacetamidophenyl R_2_, R_3_, R_4_ = 4-tolyl	Covalent (thioether)	[74,75]
**26**	Ac-NAEASAESAY-NH_2_	Porphyrin R_1_ = 4-aminophenyl R_2_, R_3_, R_4_ = phenyl	Covalent (amide)	[76]
**27**	(**a**) Ac-IQQLKNQIKQLLKQ-NH_2_ (**b**) Ac-IQQLKNQIKQLLKQAAIQ QLQNQIQQLLQQ-NH_2_	Porphyrin R_1_, R_2_, R_3_, R_4_ = 4-sulfonatophenyl	Noncovalent	[77,78,79]
**28**	(**a**) Ac-K(IEALEGK)_2_(IEALEHK) (IEALEGK)G-NH_2_ (**b**) Ac-Q(IAALEQK)(IAALE-4-Pal-K) (IAALEQK)_2_G-NH_2_	Protoporphyrin IX [Co]	Metal coordination	[80,81]
**29**	KKKKK	Phthalocyanine [Zn] R_8_, R_9_, R_10_ = H, R_11_ = COOH	Covalent (amide)	[82]
**30**	GAGAG-NH_2_	Porphyrin [Zn] R_1_ = 4-carboxyphenyl R_2_, R_3_, R_4_ = 4-*tert*-butylphenyl	Covalent (amide)	[83]
**31**	(**a**) GIGKFLHSAKKFGKAFVGEILNS(**b**) GIGKALHSAKKFGKAFVGEILNS	Porphyrin R_1_ = 4-carboxyphenyl R_2_, R_3_, R_4_ = phenyl	Covalent (amide)	[84]
**32**	Ac-IIIIKK-NH_2_	Porphyrin R_1_, R_2_, R_3_, R_4_ = 4-sulfonatophenyl	Noncovalent	[85,86,87]
**33**	PLG	Pyropheophorbide-α	Covalent (amide)	[88]
**34**	YVHD	Purpurin-18	Covalent (amide)	[89]
**35**	(**a**) QRLGVGFPK (**b**) QKVPHVGQK	Purpurin-18	Covalent (amide)	[90]
**36**	GTFG	Purpurin-18	Covalent (amide)	[91]
**37**	AKC	Purpurin-18	Covalent (amide)	[92]
**38**	(**a**) RRR (**b**) RRRRRRR	Purpurin-18	Covalent (amide)	[93]
**39**	KLVFF	Porphyrin R_1_ = 4-carboxyphenyl R_2_, R_3_, R_4_ = phenyl	Covalent (amide)	[94]
40	FFYSV	Protoporphyrin IX	Covalent (amide)	[95]

The first study of a peptide–porphyrin supramolecular self-assembly was reported in 2001 by Arai et al., who showed that conjugates **24a**,**b** and **25** can adopt β-sheet assemblies in aqueous trifluoroethanol [74]. A follow-up study indicated that this behavior is promoted by the hydrophobic effect between neighboring porphyrins, as, under the same conditions, the free peptides are too short to fold in a β-sheet [75]. The peculiar CD and UV/Vis spectra of conjugate **24a** suggest complex interactions between tetrapyrrole rings that stabilize the architecture. Long and shortwave shifting of the strong Cotton effect led the authors to assume an antiparallel peptide chain arrangement that dictate the side-by-side and face-to-face orientation of the porphyrins. When increased distance or steric hindrance between the tetrapyrroles is introduced, such as in conjugates **24b** and **25**, the assembled structure is less stable and shows simpler CD traces because the interactions are limited. Their analysis also confirmed that pH and solvent composition affect size and stability of the assemblies.

Dunetz et al. reported the pH-, time-, and temperature-controlled supramolecular assembly of conjugate **26**, in which the peptide bears two porphyrins **[76]**. The helical decapeptide scaffold plays a key role in the formation of extended porphyrin arrays with long-range electronic coupling and quenched fluorescence in trifluoroethanol at pH 6–8. This system was proposed as a building block for photonic nanodevices to mimic photosynthetic light-harvesting complexes. 

Kovaric et al. explored inducing α-helical folding in a short basic peptide with an anionic porphyrin (**27a**) [77]. They obtained a coiled-coil peptide structure tightly paired with the porphyrin, which also suggested a preliminary higher-order co-assembly. The assembly process was studied at the nanoscopic level using **27b**, a similar system containing a longer peptide: In the presence of NaCl and at neutral pH, the system replicated the assembling behavior of **27a** by forming irregular β-sheet nanofilaments [78]. Finally, regular mesoscale fibrils were obtained with system **27b** by taking advantages of J-type interactions in tetrakis(4-sulfonatophenyl)porphyrin at mild acidic conditions [79]. In these studies, time-dependent CD signals around 210–220 nm and 490 nm are indicative of the tetrapyrrole-induced coiled conformation of the peptide and the peptide-induced chiral order of the porphyrin aggregates.

Complex **28a** self-assembled into rod-like architectures with mm-scale lengths and μm-scale diameters in phosphate buffer [80]. Carvalho et al. highlighted the importance of the solvent and of the peptide–porphyrin synergy in the process, by showing that rods are not formed from the assembly of either component alone, nor from peptide–porphyrin mixtures dissolved in organic buffers or pure water. Comparing their results with similar cis-coordinated peptides, the authors postulate that the His coordination to the *trans*-axial position of the cobalt protoporphyin IX may be the reason for the linear geometry and the unidimensional morphology of the final material. Zaytsev et al. exploited a similar strategy to obtain complex **28b** in which two 30-residue coiled-coil peptides are axially coordinated via a 4-pyridylalanine residue to the cobalt in the porphyrin [81]. In water, this complex forms nanomaterials with globular shape while rods with mm-scale lengths and μm-scale diameters are observed in phosphate buffer. The conformational and assembly properties of the peptide are predominant when the globular material is formed, whereas phosphate ion creates different interactions leading to a larger and more organized morphology.

The self-assembling behavior of pentalysine-phthalocyanine **29** was instrumental to overcome the short half-life in vivo, photobleaching and toxicity displayed by stand-alone photosensitisers [82,96]. Nanodots formed in dimethyl sulfoxide/water are stable and inert in physiological conditions because the tetrapyrroles are located within a shell of positively charged polypeptides. The positive surface also directs them towards negatively charged cancer cells and makes their size pH-dependent: The dots are smaller at the acidic pH associated with tumor tissues and their cellular uptake is facilitated. Once the nanodots reach mitochondria and lysosomes, they disassemble to generate fluorescent and photodynamically-active monomers that can detect tumor or suppress its growth.

The solvent-dependent self-assembly of conjugate **30** was investigated by Wang et al. [83]. The solvent molecules determine the peptide secondary structure that, in turn, has an influence on the porphyrin packing and supramolecular chirality of the system. Specifically, *n*-hexane/tetrahydrofuran initially led to α-helix folding and then nanofibers assembling, while β-sheet and nanotubes are formed in water/tetrahydrofuran. CD analysis led the authors to confirm the presence of J-aggregates whose bisignate Cotton effects indicated the presence of right-handed chirality and left-handed chirality in the assemblies formed in hexane and water, respectively. Good conductive capabilities are the results of these highly ordered systems.

The influence of single residue substitution on the self-assembly properties of a 23-amino acid peptide–porphyrin conjugate has also been reported [84]. Conjugates **31a**,**b** were synthesized by coupling the porphyrin with two peptides bearing either A or F as the fifth residue of the sequence. Interestingly, although their secondary structure is similar, only conjugate **31a** self-assembled into micrometric fibrils in water/methanol.

A self-assembling amphiphilic peptide was exploited by Wang et al. as a template for the co-assembly of system **32** [85]. The porphyrin-functionalized β-sheet nanofilaments obtained behaved as artificial photo-responsive synthetic cell-like structures and were explored for the development of artificial photosynthetic devices [86]. The self-assembly of the peptide was not affected by the presence of the porphyrin but the formation of the porphyrin J-aggregates was favored by the peptide assemblies in mildly acidic environment and by specific peptide–porphyrin molar ratio. Initially, the encapsulation of nanofilaments within silica nanoparticle-stabilized colloidosomes lead to a photocatalytic system with high light sensitivity but low sustainability in photocatalysis reaction. The self-metallization of platinum salts after the assembly improved sustainability under light irradiation [87].

Li et al., Lin et al., and Cai et al. reported a series of in situ supramolecular chemistry strategies for self-assembling in living systems (in vitro and in vivo) [88,89,90,91,92]. They exploited targeting motif (i.e., vancomycin, TAT cell-penetrating peptide, mannose, and poly(amidoamine) dendrimers) to selectively direct conjugates to bacterial cells or macrophages. Cleavage of the enzyme responsive peptide linker and self-assembly of the remaining peptide-tetrapyrrole conjugates **33**–**37** were then monitored with CD. These systems have enhanced photoacoustic signal and are applicable for quantitative detection and real-time monitoring of bacterial infection and autophagy. Most recently, Zhou et al. evaluated the in vivo self-assembling efficiency of conjugate **38a** exploiting non-invasive and real-time photoacoustic tomography (PAT) [93]. Conjugate **38b** also self-assemble in DMSO/water but the disappearance of CD signals at higher temperature and lower amount of “bad solvent” means that its assembly is driven by weaker interactions.

Liu et al. conjugated the Aβ-targeting peptide KLVFF with a porphyrin to obtain a self-assembling photoactive system for in vivo prevention of Aβ-aggregation and Alzheimer Disease treatment [94]. Nanospheres of conjugate **39** are photothermally active and can permeate the blood–brain barrier (BBB), while their photodynamic activity is exploited for Aβ-selective photooxygenation after Aβ-triggered disassembly.

The “all-in-one self-delivery nanosystem” for tumor PAI and PTT presented by Ren et al. is a covalent conjugate of a self-assembling peptide (FF), an anticancer peptide (YSV) and a photosensitiser (Protoporphyrin IX) [95]. After injection in mice, conjugate **40** supramolecular nanorods accumulate in the tumor and, upon irradiation, the porphyrin photothermal activity, combined with the histone deacetylase inhibitor effect of the YSV motif, causes apoptosis.

## 5. Peptides Containing Non-Amino Acid Components

Table 4 shows peptides bearing non-amino acid moieties that were used in combination with tetrapyrroles to produce supramolecular self-assembled structures. The presence of long aliphatic chains, of peptide N- or C-termini protecting groups or other moieties drastically impacts the self-assembly behavior of the system.

**Table 4:** Conjugates, complexes and noncovalent assembling systems of peptides with non-amino acid components and tetrapyrroles. Refer to Figure 1 and Table S1 for details on structure and further information. [X] = metal atoms coordinated by the tetrapyrrole ring; c16 = hexadecanoic acid; c14 = tetradecanoic acid; Cbz = benzyloxycarbonyl; Aib = aminoisobutyric acid; Cha = cyclohexylalanine; NDI = naphthalene diimide; Thy = thymine; Ade = adenine.

Entry	Peptide	Tetrapyrrole	Interaction (Linker/Bond)	Ref.
**41**	(**a**) c16-AHLLLKKK (**b**) c16-AHALLLKKK (**c**) c16-AHWWWKKK (**d**) c16-AHFFFKKK (**e**) c16-AHIIIKKK (**f**) c16-AHVVVKKK (**g**) c16-AHAAAKKK	Protoporphyrin IX [Zn]	Metal coordination	[97,98,99,100]
**42**	(**a**) c16-AALLLKKK (**b**) c16-AHLLLKKK (**c**) c16-HHLLLKKK (**d**) c16-MHLLLKKK	Protoporphyrin IX [Fe]	Metal coordination	[101]
**43**	(**a**) c16-AHLLLKKK (**b**) c16-AHLLLKKKKKKKKK	Porphyrin [Zn] R_1_, R_3_ = phenyl R_2_, R_4_ = H	Metal coordination	[102]
**44**	(**a**) c14-FFK (**b**) c14-FK (**c**) c14-YYK (**d**) c14-YK	Porphyrin [Zn] R_1_, R_2_, R_3_, R_4_ = 4-pyridyl	Noncovalent	[103]
**45**	(**a**) Boc-II (**b**) Fmoc-II (**c**) Cbz-II (**d**) II-OMe	Porphyrin (a, b, c) R_1_ = 4-aminophenyl (**d**) R_1_ = 4-carboxyphenyl R_2_, R_3_, R_4_ = phenyl	Covalent (amide)	[104]
**46**	(**a**) Boc-AI (**b**) Fmoc-AI (**c**) Cbz-AI (**d**) AI-OMe	Porphyrin (a, b, c) R_1_ = 4-aminophenyl (**d**) R_1_ = 4-carboxyphenyl R_2_, R_3_, R_4_ = phenyl	Covalent (amide)	[104]
**47**	Fmoc-TL-NH_2_	Porphyrin R_1_, R_2_, R_3_, R_4_ = 4-carboxyphenyl	Noncovalent	[105]
**48**	Fmoc-LLL-OMe	Porphyrin R_1_, R_2_, R_3_, R_4_ = phenyl	Noncovalent	[106]
**49**	Fmoc-LLL-OMe	Porphyrin R_1_, R_2_, R_3_, R_4_ = 4-hydroxyphenyl	Noncovalent	[107,108]
**50**	Cbz-HF	Chlorin e6 [Zn]	Metal coordination	[109]
**51**	(**a**) Fmoc-ChaChaGK-NH_2_ (**b**) Fmoc-FFGK-NH_2_ (**c**) Ac-ChaChaGK-NH_2_ (**d**) Ac-FFGK-NH_2_	Porphyrin R_1_, R_2_, R_3_, R_4_ = 4-sulfonatophenyl	Noncovalent	[110,111]
**52**	(**a**) GGK(Biotin)-COOH (**b**) GGK(Biotin)-CONH_2_	Phthalocyanine [Zn] R_8_, R_9_, R_10_ = H R_11_ = 4-(3-propanoyl)phenoxy	Covalent	[112]
**53**	Thy-AAibAAibAAibAAib-Ade	Porphyrin R_1_ = 4-aminophenyl R_2_, R_3_, R_4_ = phenyl	Noncovalent	[113]
**54**	Ac-VE(NDI)VKVE(NDI)V-NH_2_	PorphyrinR_1_ = 4-carboxyphenyl R_2_, R_3_, R_4_ = phenyl	Covalent (amide)	[114]
**55**	(**a**) KK (**b**) KKKK (**c**) KKKKKKKK (**d**) KKKKKKKKKKKKKKKK	Porphyrin R_1_ = 4-[(4-butanoyl)oxy]phenyl R_2_, R_3_, R_4_ = 1,3-di-*tert*-butylphenyl	Covalent (amide)	[115,116]

### 5.1. Fatty Acids

**Figure 5:**
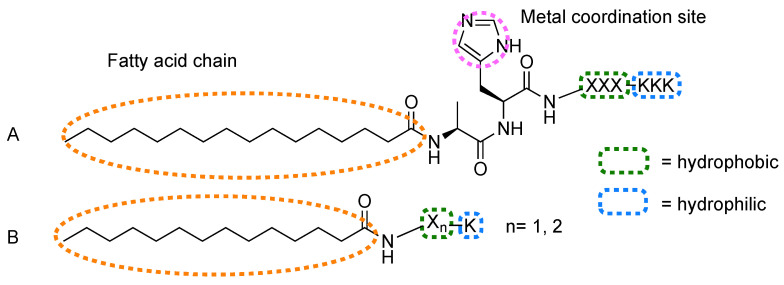
General structure of fatty acid peptide derivatives used by (**A**) Fry et al. [97,98,99,100,101,102] and (**B**) Tao et al. [103] in combination with tetrapyrroles to induce assembly.

Fry et al. used fatty acid-bearing peptides with general sequence c16-AHX_3_K_3_ (Figure 5A), where X is a hydrophobic amino acid (e.g., A, I, L, V, F, V) and H is a coordination site for Zn or Fe porphyrins, as scaffolds to obtain light harvesting fibers. 

Complex **41a** self-assembles into fibers by propagation of β-sheet sub-structures in a NH_4_OH aqueous solution at pH 11 [97]. An optimal 1:6 peptide/porphyrin stoichiometry, determined from CD analysis, is necessary to obtain fibers with well-ordered, closely packed and interacting chromophores along their axis. The authors achieved precise control over the system morphology and porphyrin organization, starting from the incorporation of both Zn and Fe protoporphyrin IX [97,101]. The addition of an alanine to the peptide (**41b**) does not influence the morphology of the self-assembled structure in basic solution but prevents exciton coupling. Changes in pH determine morphology changes in complex **41a**: At neutral pH, the peptides adopt a random coil conformation and assemble into micelles. This less-ordered system contains a mixture of mostly isolated, uncoordinated and coordinated porphyrin molecules allowing for triplet formation. The authors showed that, in both micelles and fibers, triplet yield and lifetime can be increased by reducing the concentration of chromophores to effectively separate neighboring molecules [98]. Similarly, in the nanofiber assembly of complex **41a**, the peptide–chromophore ratio can influence the packing and lead to long-range exciton transport or rapid excimer formation energy flows [99]. The LLLKKK string, responsible for the β-sheet formation and the organization of the peptide around the chromophore, was modified by replacing the L residues with three aromatic or aliphatic amino acids (complex **41c**–**g**): This affected the dissociation constant of the system and modulated the degree of interchromophore interactions [100]. Similarly, when the dialanine motif was replaced by methionine or histidine, the self-assembled structures of **42a**–**d** changed [101]. The modulation of these environmental and structural parameters ultimately allows the control over the catalytic properties of the material and their application as dye-sensitized semiconductors. Hybrid materials obtained by TiO_2_ mineralization of **43a**,**b** self-assembled fibers were able to induce charge separation upon irradiation [102]. The number of lysine residues in **43a,b** influences the degree of charge separation and the sites available for TiO_2_ mineralization. These results further emphasize the importance of controlling the morphology of the self-assembled structure for the modulation of its properties, especially where chromophore organization and concentration are key factors.

Tao et al. reported a co-assembled system based on peptide amphiphiles (Figure 5B) and porphyrins (**44a**–**d**) [103]. c14-FFK peptide alone self-assembled into wide, slightly left-handed nanoribbons, while narrow, intensely right-handed nanofibers are formed with the removal of one phenylalanine (c14−FK). When the chromophores were added, these were chirotropically organized within the unaffected nanostructures. When replacing the amino acids with their d-type enantiomers, the chiral features and corresponding CD signals were reversed, which means that the peptide handedness dictates chirality in porphyrin aggregates.

### 5.2. Protecting Groups

**Figure 6:**
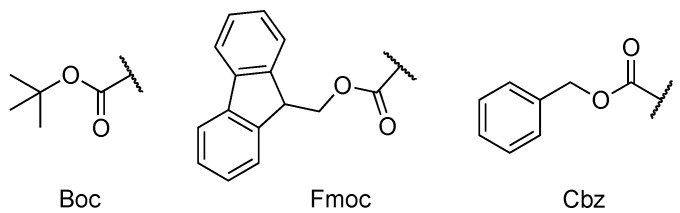
Chemical structure of Boc (tert-butyloxycarbonyl), Fmoc (fluorenylmethoxycarbonyl) and Cbz (benzyloxycarbonyl) protecting groups.

Amino acid *N*-terminal protecting groups (Figure 6) are simple peptide modifications that can affect the way conjugates and noncovalent systems self-assemble.

Fmoc/Boc protection did not particularly affect the assembly of FF (e.g., in conjugates **1a**–**c** [52,53]) but had a remarkable impact for peptides that do not self-assemble. “Spiky spheres”, “flake-shape” structures and a hydrogel were observed after the assembly of protected aliphatic dipeptides conjugates (**45a**–**d** and **46a**–**d**) in hexafluoroisopropanol/water [104]. In the same solvent mixture, the noncovalent combination of a Fmoc-protected FF with a porphyrin (**15**) also forms a hydrogel [64].

Nanofiber-based hydrogels with energy transfer capabilities are formed from the co-assembly of **47** [105]. Interestingly, the authors employed a reversible biocatalytic protocol in which thermolysin couples Fmoc-T and l-NH_2_ in aqueous solution. Afterwards, CD suggests that Fmoc dipeptides assemble in β-sheet fibers and incorporate porphyrin excitonically coupled with fluorenyl groups with a helical arrangement. At 0.2 mM concentration and peptide–porphyrin ratio of 1:100, the energy transfer efficiency in the self-assembled system is 10% while the highest value (40%) was obtained at 2 mM concentration.

A hybrid hydrogel for solar energy conversion was also obtained by Feng et al. [106]. After subtilisin-mediated methoxyl group deprotection, system **48** co-assembles into a hydrogel of protected aromatic tripeptides β-sheet nanofilaments doped with tetrapyrrole rings. The long-range ordered porphyrin can harvest solar light to produce photoelectrons while their combination with the assembled peptide nanoarchitecture grants a good electron acceptor and conductor support, yielding a final efficient photoelectron transfer. Interestingly, all the examples of hydrogel supramolecular structures involve Fmoc-protected di- or tripeptides. 

The same peptide sequence was exploited in combination with a different porphyrin derivative (**49**) to obtain co-assembled nanospheres for PDT [107,108]. Using a one-step co-assembly method in aqueous media, hydrophobic porphyrins localize as monomeric molecules on the nanoparticle surface. In this architecture, self-aggregation and fluorescent quenching of tetrapyrroles is avoided. This is advantageous for the photosensitizer delivery, for the production of ROS and for photodynamic tumor eradication in vitro and in vivo. Comparable assembly and antitumor properties were observed for **50** [109]. The resulting self-assembled spherical metal-nanodrugs showed remarkable loading capacity, encapsulation efficiency, stability under normal physiological conditions, responsivity to pH and glutathione level variations, blood circulation lifetime, accumulation in cancer cell, targeted burst release and antitumor efficacy with negligible toxicity.

Xiu et al. compared the co-assembly of tetrakis(4-sulfonatophenyl)porphyrin and Pt particles with similar peptides (**51a**–**d**) to understand how the peptide molecular structure influences the light harvesting system [110,111]. The Fmoc group and the Cha residue (**51a**) promoted the assembling and the formation of J-aggregates, enhancing the photo-to-electric conversion efficiency and photocatalytic ability (NAD+/NADH conversion) of these architectures.

### 5.3. Other Biomolecules

The presence of biotin in conjugates **52a**,**b** can direct the PDT action of self-assembled nanospheres to target cells overexpressing biotin receptor [112]. Complex **52a** can also co-assemble with the chemotherapeutic agent doxorubicin to form spherical nanoparticles that induce apoptosis in a synergistic manner and have a strong tumor inhibition effect upon irradiation in vitro and in vivo.

**Figure 7:**
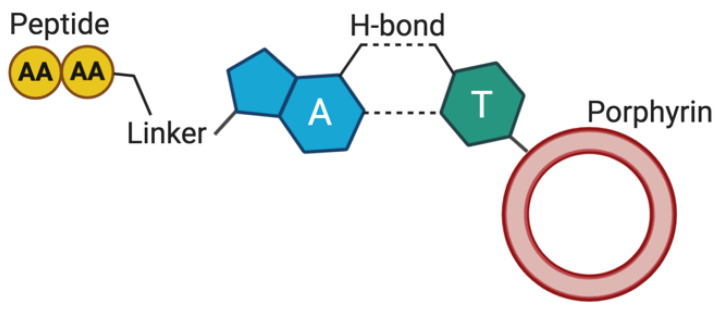
Complementary recognition between peptide-adenine (A) and porphyrin-thymine (T) in system **53**. Created with Biorender.com.

Assemblies of peptide nucleic acid (PNA) with and without tetrapyrroles are reported in the literature [117], but only one example is available describing the co-assembly of a nucleobase-containing peptide with a tetrapyrrole ring (**53**) [113]. As part of a broader study aimed at controlling the peptide assembly via thymine-adenine complementary recognition, Marafon et al. observed that an α-aminoisobutyric acid-rich helical peptide, containing the two nucleobases covalently linked at the N- and C-termini, assembles with porphyrin-derivatized thymine (Figure 7). In tetrahydrofuran/water, vesicles with variable size and distribution depending on the molar ratio of the two components are formed, while the addition of trifluoroethanol promotes the transition from supramolecular vesicles to the fiber morphology.

### 5.4. Electron Acceptor Units

The addition of electron acceptor moieties to peptide-tetrapyrrole systems provides an interesting approach to develop new electronically and optically active materials. The self-assembling behavior of conjugate **54**, which bears two electron-accepting naphthalene diimides (NDI) covalently attached to the heptapeptide side chains, was characterized by Nakayama et al. [114]. In trifluoroethanol-chloroform/hexafluoroisopropanol mixtures, β-sheet fibers are formed when the porphyrin is in the neutral form, while its protonation prevents the adoption of any secondary structure.

Hasobe et al. studied the self-assembly of K-based peptides of various lengths bearing multiple porphyrins [115,116]. They synthesized a porphyrin-derivatized lysine that was subsequently coupled to form dimers, tetramers, octamers and hexadecamers (conjugates **55a**–**d**) [118]. The peptide oligomers self-assembled with fullerene onto a nanostructured SnO_2_ electrode to form supramolecular clusters that were employed for the design and fabrication of organic photovoltaic cells.

## 6. Cyclic Peptides

The relatively flat conformation of many cyclic peptides (CP) favors their organization into nanotubular assemblies through parallel stacking of different monomers. Despite a prevalence of literature work on such peptide architectures [13], there are only a few examples of cyclic peptides assembling with tetrapyrroles. Unlike linear peptides, the reported cyclopeptides conjugated with tetrapyrroles exceptionally leads to dimer formation and not to long-range ordered organizations. As shown in Table 5, these are based on α,γ-cyclic peptides described by Granja-Ballester group.

**Table 5:** Cyclic peptide-tetrapyrrole conjugates. Refer to Figure 1 and Table S1 for details on structure and further information. [X] = Metal atoms coordinated by the tetrapyrrole ring. Ach = Cyclohexane; Acp = Cyclopentane.

Entry	Peptide	Tetrapyrrole	Interaction (Linker/Bond)	Ref.
**56**	c[(S-d-^Me^N-γ-Ach-F-d-^Me^N-γ-Ach)_2_]	Porphyrin [Zn] R_1_ = 4-carboxyphenyl R_2_, R_3_, R_4_ = 4-pentylphenyl	Covalent (ester)	[119]
**57**	c[S-d-^Me^N-γ-Acp-(F-d-^Me^N-γ-Acp)_2_]	Porphyrin [Zn] R_1_ = 4-carboxyphenyl R_2_, R_3_, R_4_ = 4-pentylphenyl	Covalent (ester)	[120]
**58**	c[(I-^Me^N-γ-Acp-I-^NHNHAc^N-γ-Acp-)_2_]	Porphyrin [Zn] R_1_, R_3_ = 3-formylphenyl R_2_, R_4_ = mesityl	Covalent (hydrazone)	[121]

**Figure 8:**
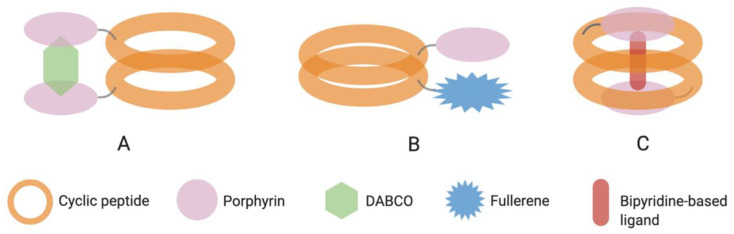
Graphical representation of conjugates **56** (**A**), **57** (**B**), and **58** (**C**) dimers. Created with BioRender.com.

Conjugate **56**, a cyclic octapeptide bearing one or two Zn–porphyrin units, forms different rotational isomers of dimeric assemblies in solution [119]. However, two adjacent porphyrin moieties offer a ditopic coordination site with a third component, 1,4-diazabicyclo[2.2.2]octane (DABCO), that regioselectively directs the process towards the formation of sandwich-like complex and a final co-facial assembly (Figure 8A). Aragay et al. thermodynamically characterized similar homo and heterodimers of conjugate **57** [120]. Attractive interactions and photoinduced electron transfer between the electron donor Zn porphyrin and electron acceptor fullerene were reported in the energetically preferred eclipsed conformation of the tubular heterodimer shown in Figure 8B. Conjugate **58** forms a singular system by self-assembling into a dimeric molecular capsule that is able to selectively recognize and encapsulate a guest molecule (bipyridine-based ligands of suitable sizes) by metal coordination [121]. In the dimer (Figure 8C), the porphyrins are not directly involved in the assembly, which is directed by the cyclic peptide, but are rather exploited as caps for the molecular cage. The peptide–porphyrin hydrazone connection is then acting as dynamic covalent bond to liberate the encapsulated ligand after hydrolysis.

## 7. Conclusions and Outlook

The last two decades brought dramatic advances in the field of self-assembled systems comprising tetrapyrroles and self-assembling peptides. This led to new supramolecular architectures that allow exploiting in an unprecedented way the photophysical and photochemical properties of tetrapyrroles. Novel materials have been created, whose 3D morphologies and long-range ordered architectures can be tuned by the design of the molecular building blocks and by careful adjustment of the environment conditions they are generated in. The studies reviewed here demonstrate how the morphology of the assembly has a major impact on the properties and behavior of the tetrapyrrole in the resulting self-assembled material. As a result, thorough understanding of the assembly mechanism is paramount to enable control of the resulting supramolecular architectures and optimize their properties for the target applications. These bioinspired supramolecular assemblies showed a great potential as delivery tools for photosensitisers, as catalysts and as biomimetic light harvesting systems. Combinations of tetrapyrroles that have been less explored for self-assembly (i.e., phthalocyanines, corroles and porphycenes) with self-assembling peptides such as cyclic or bolaamphiphiles species will further expand the library of building blocks to generate innovative light-responsive soft materials.

## Supplementary Materials

The following are available online, Table S1: Peptide–tetrapyrroles conjugates, complexes, and noncovalent assembling systems.
