# Supplementary material for: Peptide-Tetrapyrrole Supramolecular Self-Assemblies: State of the Art
Source: Molecules. 2021 Jan 28;26(3):693. doi: 10.3390/molecules26030693 (PMC7865683; doi:10.3390/molecules26030693)
Supplement: Supplementary file 1 [file molecules-26-00693-s001.pdf]

Supplementary Materials

Peptide-Tetrapyrrole Supramolecular Self-Assemblies: State of the Art

Paolo Dognini [1], Christopher R. Coxon [2], Wendel A. Alves [3] and Francesca Giuntini [1,*]

[1] School of Pharmacy and Biomolecular Sciences, Byrom Street Campus, Liverpool John Moores University, Liverpool, L3 3AF, UK; P.Dognini@ljmu.ac.uk
[2] Institute of Chemical Sciences, School of Engineering and Physical Sciences, Heriot-Watt University, Edinburgh, EH14 4AS, UK; C.Coxon@hw.ac.uk
[3] Centro de Ciências Naturais e Humanas, Universidade Federal do ABC, 09210-380, Santo André, São Paulo, Brazil; wendel.alves@ufabc.edu.br
* Correspondence: F.Giuntini@ljmu.ac.uk; Tel.: +441512312072

Table S1. Peptide-tetrapyrroles conjugates, complexes and non-covalent assembling systems. For references, please refer to the main article.
DSSC= Dye Sensitised Solar Cell; PAI= Photoacoustic imaging; PTT= Photothermal therapy; PDT= Photodynamic therapy; FI= Fluorescence imaging.

Entry	Peptide	Tetrapyrrole	Interaction (linker/bond)	Structure (aggregate)	Application	Ref
**1**	(**a**) FF<br>(**b**) Boc-FF<br>(**c**) Fmoc-FF	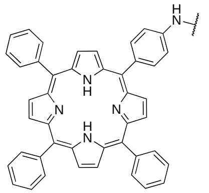	Covalent (amide)	Nanosphere<br>Microfibril<br>Platelet<br>(J-/H-aggregates)	DSSC	[52,53]
**2**	FF-OMe	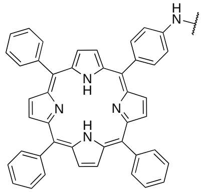	Covalent (triazine)	Nanosphere	-	[54]
**3**	Boc-FF	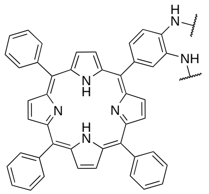	Covalent (amide)	Nanosphere	-	[54]
**4**	Boc-FF	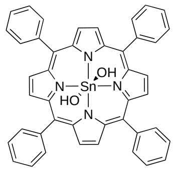	Metal coordination	Nanosphere	-	[54]
**5**	(**a**) Fmoc-FF<br>(**b**) FF-OMe	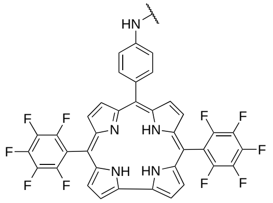	Covalent<br>(**a**) amide<br>(**b**) triazine	Nanosphere	-	[54]

Entry	Peptide	Tetrapyrrole	Interaction (linker/bond)	Structure (aggregate)	Application	Ref
**6**	Boc-FF	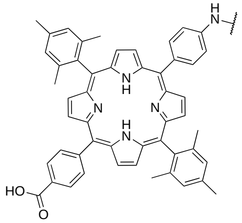	Covalent (amide)	-	DSSC	[55]
**7**	(**a**) FF<br>(**b**) Boc-FF<br>(**c**) Fmoc-FF	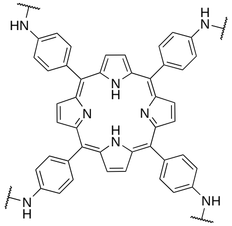	Covalent (amide)	Nanosphere<br>Microfibril<br>Plaque<br>(J-aggregate)	-	[56]
**8**	FF-BODIPY	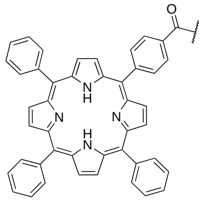	Covalent (amide)	Microsphere<br>(J-aggregate)	-	[56]
**9**	FF	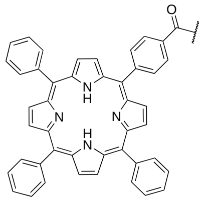	Covalent (amide)	Microsphere	Light harvesting and energy transfer<br>Photocatalysis	[57]
**10**	FF	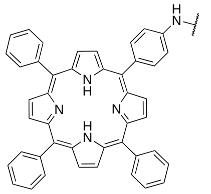	Covalent (triazine)	Nanosphere<br>Nanoparticle<br>Nanosheet<br>(J-aggregate)	Light harvesting and energy transfer	[58]

Entry	Peptide	Tetrapyrrole	Interaction (linker/bond)	Structure (aggregate)	Application	Ref
**11**	FF	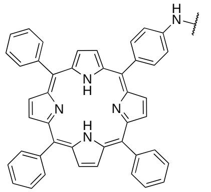	Covalent (glutaric acid)	Nanodots	PTT, PAI	[59]
**12**	FF	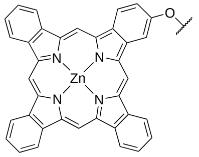	Covalent (butanoic acid)	Nanofibrils (J-aggregate)<br>Nanosphere (H-aggregate)	PTT, PAI, PDT, FI	[60,61]
**13**	FF-NH_2·HCl	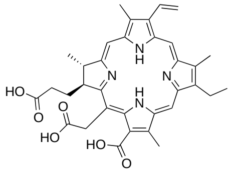	Noncovalent	Nanosphere	PDT	[62]
**14**	FF	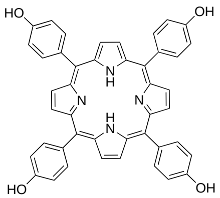	Noncovalent	Nanotube<br>(J-aggregate)	Light harvesting and energy transfer	[63]
**15**	Fmoc-FF	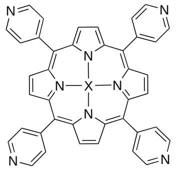<br>X= H_2, Zn, Sn	Noncovalent	Hydrogel<br>(J-aggregate)	Photocatalysis	[64]

Entry	Peptide	Tetrapyrrole	Interaction (linker/bond)	Structure (aggregate)	Application	Ref
**16**	Fmoc-FF	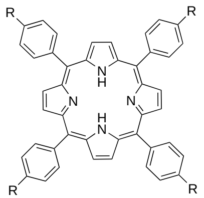<br>(**a**) R= COOH<br>(**b**) R= NH_2	Noncovalent	Hydrogel<br>(J-/H-aggregates)	Light harvesting and energy transfer	[65]
**17**	FF	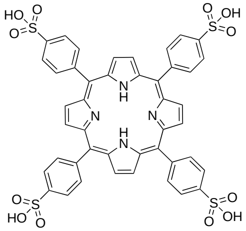	Noncovalent	Microsphere<br>(J-aggregate)	Photocatalysis	[66]
**18**	D-F-D-F-NH_2	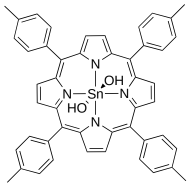	Noncovalent	Nanoribbon	DNA sensor	[67]
**19**	FF	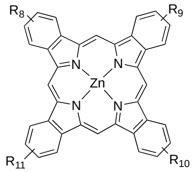<br>(**a**) R_8, R_9, R_{10}, R_{11}= 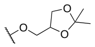<br>(**b**) R_8, R_9, R_{10}, R_{11}= 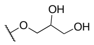<br>(**c**) R_8, R_9, R_{10}= 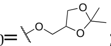; R_{11}= CH_3<br>(**d**) R_8, R_9, R_{10}= 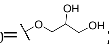; R_{11}= CH_3	Noncovalent	Sharp-edged structure	PDT	[68]

Entry	Peptide	Tetrapyrrole	Interaction (linker/bond)	Structure (aggregate)	Application	Ref
**20**	GG	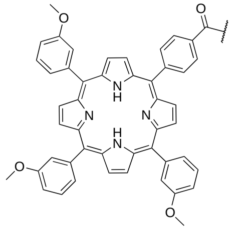	Covalent (amide)	Rod<br>Sphere<br>(J-/H-aggregate)	-	[69]
**21**	KK	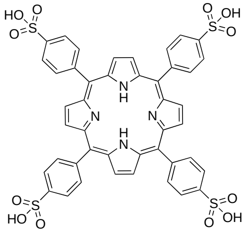	Noncovalent	Fibre<br>(J-aggregate)	Light harvesting and energy transfer<br>Photocatalysis	[70,71]
**22**	YY	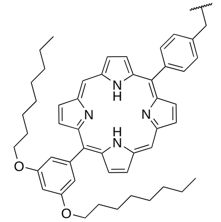	Covalent (ether)	Nanofibre<br>Toroid<br>(H-aggregate)	-	[72]
**23**	WG	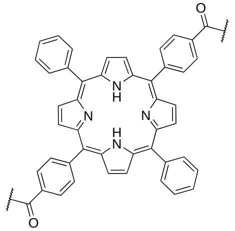	Covalent (amide)	Nanoparticle<br>Nanofibre	PDT	[73]

Entry	Peptide	Tetrapyrrole	Interaction (linker/bond)	Structure (aggregate)	Application	Ref
**24**	Ac-CKVKV-NH_2	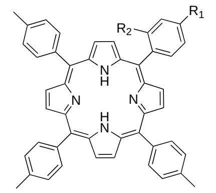 (**a**) R_1= 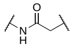 R_2= H (**b**) R_1= H R_2= 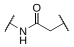	Covalent (thioether)	β-sheet	-	[74,75]
**25**	Ac-CKVSVKV-NH_2	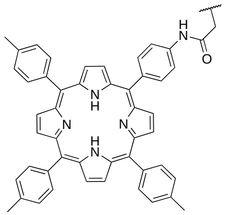	Covalent (thioether)	β-sheet	-	[74,75]
**26**	Ac-NAEASAESAY-NH_2	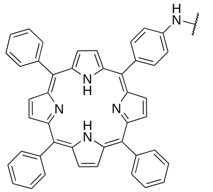	Covalent (amide)	Extended array (J-aggregate)	Light harvesting and energy transfer	[76]
**27**	(**a**) Ac-IQQLKNQIKQLL KQ-NH_2 (**b**) Ac-IQQLKNQIKQLLKQA AIQQLQNQIQQLLQQ-NH_2	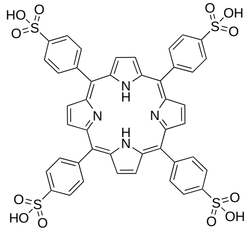	Noncovalent	Mesoscale fibrils (J-aggregate)	-	[77-79]

Entry	Peptide	Tetrapyrrole	Interaction (linker/bond)	Structure (aggregate)	Application	Ref
**28**	(**a**) Ac-K(IEALEGK)$_2$(IEALEHK)(IEALEGK)G-NH$_2$<br>(**b**) Ac-Q(IAALEQK)(IAALE-4-Pal-K)(IAALEQK)$_2$G-NH$_2$	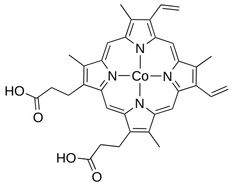	Metal coordination	Rod<br>Sphere	-	[80,81]
**29**	KKKKK	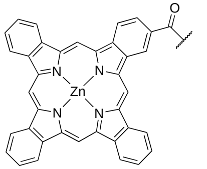	Covalent<br>(amide)	Nanodot	PDT, FI	[82]
**30**	GAGAG-NH$_2$	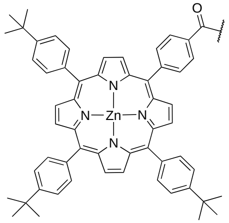	Covalent<br>(amide)	Nanofibre<br>Nanotube<br>(J-aggregate)	Semiconductor	[83]
**31**	(**a**) GIGKFLHSAKKFGKAFVGEILNS<br>(**b**) GIGKALHSAKKFGKAFVGEILNS	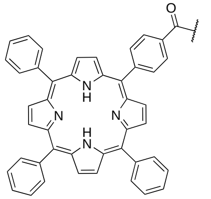	Covalent<br>(amide)	Microfibril<br>(J-aggregate)	-	[84]
**32**	Ac-IIIIKK-NH$_2$	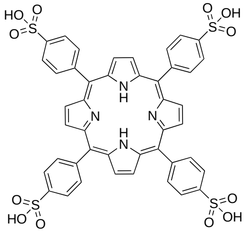	Noncovalent	Nanofibre<br>(J-aggregate)	Light harvesting and energy transfer<br>Photocatalysis	[85-87]

Entry	Peptide	Tetrapyrrole	Interaction (linker/bond)	Structure (aggregate)	Application	Ref
**33**	PLG	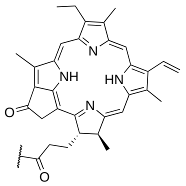	Covalent (amide)	Fibre	PAI	[88]
**34**	YVHD	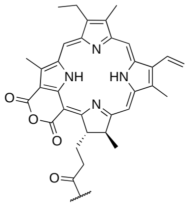	Covalent (amide)	Fibre	PAI	[89]
**35**	(**a**) QRLGVGFPK<br>(**b**) QKVPHVGQK	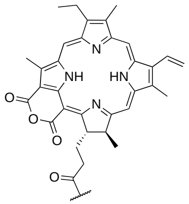	Covalent (amide)	Nanoparticle	-	[90]
**36**	GTFG	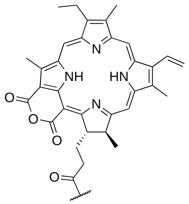	Covalent (amide)	Nanofibre	PAI	[91]
**37**	AKC	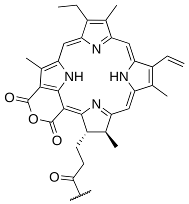	Covalent (amide)	Fibre (J-aggregate)	PAI	[92]

Entry	Peptide	Tetrapyrrole	Interaction (linker/bond)	Structure (aggregate)	Application	Ref
**38**	(**a**) RRR<br>(**b**) RRRRRRR	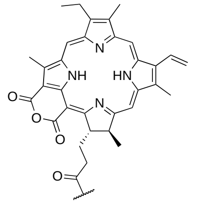	Covalent<br>(amide)	Fibre<br>(J-aggregate)	PAI	[93]
**39**	KLVFF	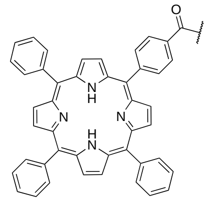	Covalent<br>(amide)	Nanosphere	Prevention of Aβ aggregation	[94]
**40**	FFYSV	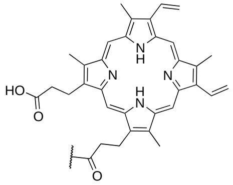	Covalent<br>(amide)	Nanorod	PTT, PAI	[95]
**41**	(**a**) c16-AHLLLKKK<br>(**b**) c16-AHALLLKKK<br>(**c**) c16-AHWWWKKK<br>(**d**) c16-AHFFFKKK<br>(**e**) c16-AHIIIKKK<br>(**f**) c16-AHVVVKKK<br>(**g**) c16-AHAAAKKK	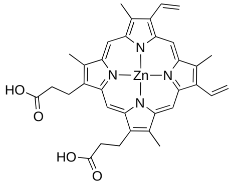	Metal coordination	Fibre<br>Micelle	Light harvesting and energy transfer<br>Photocatalysis	[97-100]
**42**	(**a**) c16-AALLLKKK<br>(**b**) c16-AHLLLKKK<br>(**c**) c16-HHLLLKKK<br>(**d**) c16-MHLLLKKK	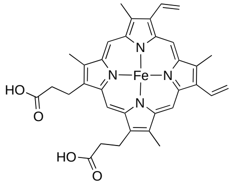	Metal coordination	Fibre<br>Micelle	Photocatalysis	[101]

Entry	Peptide	Tetrapyrrole	Interaction (linker/bond)	Structure (aggregate)	Application	Ref
**43**	(**a**) c16-AHLLLKKK<br>(**b**) c16-AHLLLKKKKKKKKK	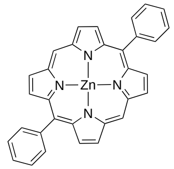	Metal coordination	Fibre	DSSC<br>Photocatalysis	[102]
**44**	(**a**) c14-FFK<br>(**b**) c14-FK<br>(**c**) c14-YYK<br>(**d**) c14-YK	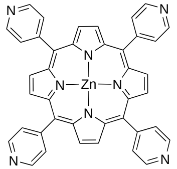	Noncovalent	Nanoribbon<br>Nanofibre<br>(J-aggregate)	-	[103]
**45**	(**a**) Boc-II<br>(**b**) Fmoc-II<br>(**c**) Cbz-II<br>(**d**) II-OMe	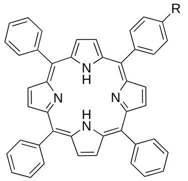<br>(**a**, **b**, **c**) R= NH_2<br>(**d**) R= COOH	Covalent<br>(amide)	Spheres<br>Flakes<br>Spikes<br>(J-aggregate)	-	[104]
**46**	(**a**) Boc-AI<br>(**b**) Fmoc-AI<br>(**c**) Cbz-AI<br>(**d**) AI-OMe	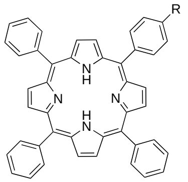<br>(**a**, **b**, **c**) R= NH_2<br>(**d**) R= COOH	Covalent<br>(amide)	Sphere<br>(J-aggregate)	-	[104]

Entry	Peptide	Tetrapyrrole	Interaction (linker/bond)	Structure (aggregate)	Application	Ref
**47**	Fmoc-TL-NH_2	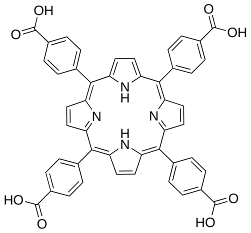	Noncovalent	Hydrogel	Light harvesting and energy transfer	[105]
**48**	Fmoc-LLL-OMe	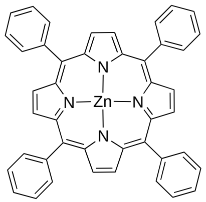	Noncovalent	Hydrogel (J-aggregate)	Light harvesting and energy transfer	[106]
**49**	Fmoc-LLL-OMe	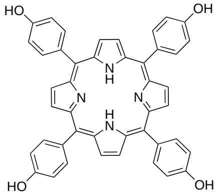	Noncovalent	Nanoparticle	PDT	[107, 108]
**50**	Cbz-HF	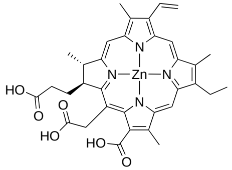	Metal coordination	Nanosphere	PDT	[109]
**51**	(**a**) Fmoc-ChaChaGK-NH_2<br>(**b**) Fmoc-FFGK-NH_2<br>(**c**) Ac-ChaChaGK-NH_2<br>(**d**) Ac-FFGK-NH_2	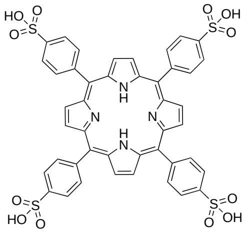	Noncovalent	Nanoribbons<br>Nanorods	Light harvesting and energy transfer	[110, 111]

Entry	Peptide	Tetrapyrrole	Interaction (linker/bond)	Structure (aggregate)	Application	Ref
**52**	(**a**) GGK(Biotin)-COOH<br>(**b**) GGK(Biotin)-CONH$_2$	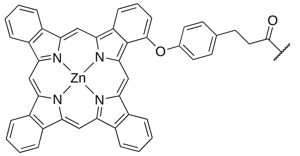	Covalent<br>(amide)	Nanosphere	PDT	[112]
**53**	Thy-AAibAAibAAibAAib-Ade	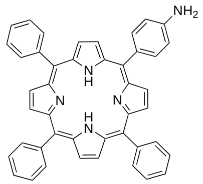	Noncovalent	Vesicle<br>Fibre	-	[113]
**54**	Ac-VE(*NDI*)VKVE(*NDI*)V-NH$_2$	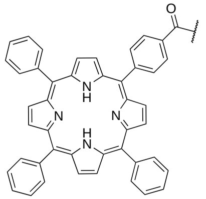	Covalent<br>(amide)	Fibre<br>(J-/H-aggregate)	-	[114]
**55**	(**a**) KK<br>(**b**) KKKK<br>(**c**) KKKKKKKK<br>(**d**) KKKKKKKKKKKKKKKK	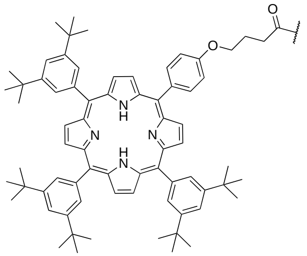	Covalent<br>(amide)	Cluster	DSSC	[115, 116]
**56**	c[(S-D-^{Me}N-γ-Ach-F-D-^{Me}N-γ-Ach)$_2$]	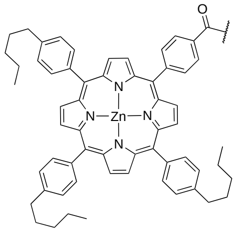	Covalent (ester)	Dimer	-	[119]

Entry	Peptide	Tetrapyrrole	Interaction (linker/bond)	Structure (aggregate)	Application	Ref
**57**	c[S-D-^{Me}N-γ-Acp-(F-D-^{Me}N-γ-Acp)$_2$]	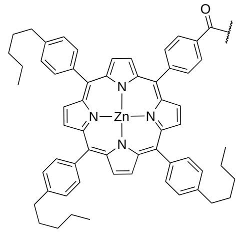	Covalent (ester)	Dimer	-	[120]
**58**	c[(I-^{Me}N-γ-Acp-I-^{NHNHAc}N-γ-Acp-)$_2$]	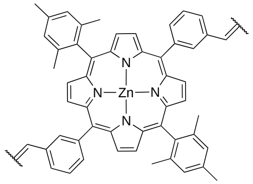	Covalent (hydrazone)	Dimer	Molecular capsule	[121]
